# Immunosuppressive effects of radiation on human dendritic cells: reduced IL-12 production on activation and impairment of naïve T-cell priming

**DOI:** 10.1038/sj.bjc.6602518

**Published:** 2005-04-05

**Authors:** A Merrick, F Errington, K Milward, D O'Donnell, K Harrington, A Bateman, H Pandha, R Vile, E Morrison, P Selby, A Melcher

**Affiliations:** 1 Cancer Research UK Clinical Center, St James's University Hospital, Beckett Street, Leeds LS9 7TF, UK; 2 Institute of Cancer Research, Chester Beatty Laboratories, London SW3 6JB, UK; 3 Somers Cancer Research Building, Southampton General Hospital, Southampton SO16 6YD, UK; 4 Department of Oncology, St George's Hospital Medical School, London SW17 0RE, UK; 5 Molecular Medicine Program, Mayo Clinic, Rochester, MN 55905, USA

**Keywords:** immunotherapy, cytotoxic T cells, cytokines, interleukin-12

## Abstract

Dendritic cells (DC) are professional antigen-presenting cells (APC) of the immune system, uniquely able to prime naïve T-cell responses. They are the focus of a range of novel strategies for the immunotherapy of cancer, a proportion of which include treating DC with ionising radiation to high dose. The effects of radiation on DC have not, however, been fully characterised. We therefore cultured human myeloid DC from CD14^+^ precursors, and studied the effects of ionising radiation on their phenotype and function. Dendritic cells were remarkably resistant against radiation-induced apoptosis, showed limited changes in surface phenotype, and mostly maintained their endocytic, phagocytic and migratory capacity. However, irradiated DC were less effective in a mixed lymphocyte reaction, and on maturation produced significantly less IL-12 than unirradiated controls, while IL-10 secretion was maintained. Furthermore, peptide-pulsed irradiated mature DC were less effective at naïve T-cell priming, stimulating fewer effector cells with lower cytotoxicity against antigen-specific targets. Hence irradiation of DC *in vitro*, and potentially *in vivo*, has a significant impact on their function, and may shift the balance between T-cell activation and tolerisation in DC-mediated immune responses.

Dendritic cells (DC) are the most potent of antigen-presenting cells (APC), unique in their ability to prime a naïve T-cell response (Banchereau and Steinman, 1998; Guermonprez *et al*, 2002). Immature DC (IDC) acquire antigen in the periphery and, in response to a range of activation or maturation signals, migrate to lymph nodes as mature DC (MDC), where they interact with responsive T cells (Rescigno *et al*, 1997). This interaction can result in immune tolerisation or activation, depending on the phenotype and functional state of involved DC. Effective initiation of an acquired immune response requires DC which are fully activated, expressing high levels of MHC and co-stimulatory molecules, and secreting the cytokines required to prime a potent helper T-cell response (particularly IL-12) (Steinman *et al*, 2003).

The effects of ionising radiation on DC are largely unknown, with limited data available. Irradiated IDC were less efficient at stimulating T-cell proliferation, with or without antigen, in an autologous mixed lymphocyte reaction (MLR) (Anton *et al*, 1998; Cao *et al*, 2004), while a recent report showed a differential effect of irradiation on antigen presentation by mouse DC, with impairment of T-cell priming against endogenously processed antigen, but an enhanced response against exogenously pulsed peptide (Liao *et al*, 2004).

The functional consequences of irradiating human DC are important in several contexts. The ability to culture DC from blood products *ex vivo* has led to a rapid expansion in clinical immunotherapy protocols using DC to activate or nullify antigen-specific immune responses (Melcher *et al*, 2002). The majority of these approaches are for the immunotherapy of cancer, and involve loading DC with tumour-associated antigens prior to vaccination of the patient. There is a wealth of pre-clinical data to support such strategies, and early studies have shown some promise (Timmerman and Levy, 1999). Alternatively, antigen-loaded DC can be used *ex vivo* to expand specific effector T-cell populations for subsequent adoptive transfer back into patients (Yee *et al*, 2000; Szmania *et al*, 2001; Ho *et al*, 2003). During many of these applications, DC are irradiated with X-rays or gamma sources, often to high doses (typically 30 Gy) in a single fraction. In some cases, such as irradiation of DC/tumour cell co-cultures or hybrids (Gong *et al*, 1997; Celluzzi and Falo, 1998), this treatment is rationally designed to prevent any outgrowth of viable tumour cells from within the proposed vaccine. In others, such as vaccination with tumour lysate-loaded DC (Fields *et al*, 1998), and priming of T cells *in vitro* for subsequent adoptive transfer (Szmania *et al*, 2001), the purpose of DC irradiation is less clear and potentially deleterious. Regardless of application, irradiation of DC is often assumed to have no bearing on subsequent DC/T-cell interactions or immune priming and to be immunologically neutral. However, this assumption has not been rigorously tested.

Irradiating endogenous DC *in situ* within tumours or elsewhere in patients may also be functionally important. Clinical radiotherapy is regarded as being broadly immunosuppressive due to effects on sensitive immune cell target populations such as lymphocytes (Illidge, 1998), although radiation may also stimulate immunity by inducing danger signals in association with tumour cell death (McBride *et al*, 2004). Although the mechanisms underlying interaction between radiation and the immune system are poorly understood, radiotherapy has been intentionally combined with immunotherapy to enhance tumour-specific immune activation in preclinical models (Chakravarty *et al*, 1999; Teitz-Tennenbaum *et al*, 2003; Milas *et al*, 2004). Optimisation of such protocols, which rely on endogenous or *ex vivo* cultured DC for their success, may be facilitated by an improved understanding of the effects of ionising radiation on DC effector function.

Therefore, we have compared irradiated human myeloid DC with untreated controls to characterise the consequences of irradiation on *ex vivo* DC applications or potential *in vivo* treatments. We show that human DC are resistant to radiation-induced apoptosis and show few changes in phenotypic surface markers. Irradiated DC maintain their ability to take up antigen and migrate *in vitro*, but are less potent in an allogeneic MLR. Importantly, irradiated DC alter the balance of the cytokines they secrete on activation, by reducing their IL-12 production while maintaining IL-10. Irradiated DC pulsed with a tumour antigen peptide prime fewer, less potent cytotoxic T cells (CTL) than unirradiated controls. These effects of irradiation occur downstream of the translocation of RelB to the nucleus, an event associated with DC maturation. These data have implications for the application of DC both *in vitro* and *in vivo*, and suggest that direct DC irradiation should be avoided when trying to generate antigen-specific responses.

## MATERIALS AND METHODS

### Dendritic cell culture and characterisation

Dendritic cells were generated from human peripheral blood mononuclear cells (PBMC), according to protocols previously published (Pickl *et al*, 1996; Romani *et al*, 1996). Briefly, adherent or CD14^+^-selected monocytes were cultured in RPMI 1640 (Gibco BRL, Paisley, UK) supplemented with 10% v v^−1^ FCS, 1% v v^−1^ L-glutamine (Gibco BRL, Paisley, UK), 800 U ml^−1^ GMCSF (Schering-Plough, Cork, Ireland) and 500 U ml^−1^ IL-4 (R&D Systems, Abingdon, UK) for 5 days. Where indicated, DC were irradiated to 30 Gy at 2.7 Gy min^−1^ (GammaCell Elite 1000 ^137^Cs source) and maturation induced by addition for 24 h of 100 ng ml^−1^ LPS (Sigma, Poole, Dorset, UK). Anti-human HLA-DR-PE, CD80-PE, CD83-PE, CD86-PE, CD1a-PE and CCR7-PE (BD Bioscience Pharmingen, Cowley, Oxford, UK) were used for phenotypic characterisation of DC. All samples were counted and analysed using a FACSCaliber and CellQuest Pro software^©^ 2000 (Becton, Dickinson and Company, Hertfordshire, UK).

### Apoptotis assay

Apoptosis was quantified using the Annexin-V FITC apoptosis detection kit (BD Biosciences Pharmingen, Cowley, Oxford, UK), according to the manufacturer's instructions.

### Dendritic cell functional analysis: fluid phase endocytosis, phagocytosis, migration and allogeneic MLR

Endocytosis by DC was assayed by incubation with DQ™BSA (Molecular Probes Inc., Eugene, OR, USA), a quenched BODIPY® fluorescent dye-labelled molecule that releases fluorophores by intracellular hydrolysis. Relative fluorescence is quantifiable by FACS analysis (excitation and emission maxima of 505 and 515 nm, respectively).

Phagocytosis of tumour cells by DC was assessed by fluorescent labelling of tumour cells prior to co-incubation. SW480 tumour cells were dyed for 45 min at 37°C with 1 *μ*M CellTracker™ red (Molecular Probes Inc, Eugene, Oregon, USA), rinsed thoroughly, and co-cultured with DC for 4 h. The cells were viewed by fluorescent microscopy to confirm the presence of a stained population, which was quantified by FACS analysis.

Live imaging and migration of DC was assayed in 35 mm glass-bottomed dishes (Bibby Sterilin, Stone, UK) coated with 50 *μ*g ml^−1^ fibronectin (Sigma, Poole, Dorset, UK). Dendritic cells were seeded at 3 × 10^5^ per dish in pre-warmed media supplemented with 20 mM HEPES and transferred to a Zeiss Axiovert 200 inverted microscope with an enclosed, heated stage (Solent Scientific Ltd, Portsmouth, UK) with a maintained temperature of 37°C and left for 30–45 min prior to filming. Time-lapse phase-contrast images were captured using an × 20 dry lens at 15 s intervals for a duration of 30 min with a Hamamatsu Orca 2 ER camera using 2 × 2 binning and exposure times of less than 500 ms frame^−1^. Microscope, camera and shutters were controlled by Kinetic Imaging AQM 6 software. All cells remaining within the field of view for the full 30 min were tracked and analysed for distance travelled and velocity using Kinetic Imaging Motion Analysis software.

To assess allogeneic T-cell proliferation in a MLR, day 5 DC were irradiated/matured for 24 h prior to assay. Allogeneic lymphocytes (2 × 10^5^ per well) were plated in U-bottomed 96-well plates. Dendritic cells were serially diluted and added at ratios ranging from 1 : 10 to 1 : 160 DC to responding lymphocytes and co-incubated for 5 days. ^3^H-thymidine (0.5 *μ*Ci per well) was added for 18 h before harvesting (TOMTEC Harvester 96 Mach IIIM) onto filter mats and counting (Wallac Jet 1450 Microbeta scintillation counter and Microbeta windows software, Wallac, Oy Finland).

### Cytokine detection

Levels of IL-10 and IL-12 p70 were measured by ELISA using matched paired antibodies (BD Biosciences, Pharmingen, Cowley, Oxford, UK) according to the manufacturer's instructions.

### Generation of CTL and cytotoxicity assay

RPMI 1640 supplemented with 7.5% v v^−1^ FCS, 1% v v^−1^ L-glutamine, 1% v v^−1^ non-essential amino acids (Gibco BRL, Paisley, UK), 1% v v^−1^ sodium pyruvate (Gibco BRL, Paisley, UK), 1% v v^−1^ hepes (Gibco BRL, Paisley, UK) and 20 *μ*M 2-*β* mercaptoethanol was used for all CTL cultures. Irradiated or unirradiated HLA-A2+MDC were pulsed with HLA-A2-restriced MelanA/MART-1_27–35_ (AAGIGILTV) peptide for 1 h and mixed with autologous PBMCs at a ratio of 1 : 10–1 : 30. IL-7 (5 ng ml^−1^; R&D systems, Abingdon, UK) was added to cultures from day 1 and throughout; 30 U ml^−1^ IL-2 (R&D systems, Abingdon, UK) was added on day 3 only. T cells were re-stimulated twice at weekly intervals, each time with peptide-pulsed DC in identical proportions. At 21 days, cells were harvested and cytotoxicity of CTL measured using a standard 4 h ^51^Cr release assay. Briefly, T2 target cells were pulsed with 4 *μ*g ml^−1^ MART-1, irrelevant or no peptide and labelled with 100 *μ*Ci Cr^51^ for 1 h, then washed three times and co-cultured with CTL at different effector : target ratios. To reduce background/nonspecific killing, the same number of unlabelled K562 cells as target cells was added to each condition. After 4 h, cells were spun down and 50 *μ*l of supernatant was transferred to scintillation plates (Packard Biosciences, Groningen, The Netherlands) and dried overnight before counting. Percentage lysis was calculated using the following formula:

% lysis=100 × (sample cpm−spontaneous cpm)/(maximum cpm−spontaneous cpm).

### Nuclear extracts and Western blotting

Nuclear extracts of DC were prepared as described previously (Schreiber *et al*, 1989). Briefly, DC were washed with PBS and resuspended at a maximum of 1 × 10^6^ cells in 400 *μ*l of cold buffer A (10 mM HEPES, pH 7.9; 10 mM KCl; 0.1 mM EDTA; 0.1 mM EGTA; 1 mM DTT; 0.5 mM PMSF). The cells were allowed to swell for 15 min on ice, before adding 25 *μ*l of 10% Igepal solution. The tubes were vortexed thoroughly, centrifuged and the supernatant removed. The nuclear pellet was resuspended in 50 *μ*l cold buffer B (20 mM HEPES, pH 7.9; 0.4 M NaCl; 1 mM EDTA; 1 mM EGTA; 1 mM DTT; 1 mM PMSF) and vigorously shaken for 15 min at 4°C before centrifugation. The soluble nuclear fraction was removed, aliquoted and stored at −80°C. Nuclear protein concentration was determined by Bradford Assay and 10 *μ*g protein samples were resolved by 10% SDS–polyacrylamide gel electrophoresis before transfer onto nitrocellulose membrane. The blots were probed with anti-RelB rabbit polyclonal antibody (Santa Cruz, CA, USA) and horseradish peroxidase-conjugated secondary antibody (Dako Cytomation, CA, USA) before visualisation with SuperSignal West Pico Chemiluminescent Substrate (Pierce Biotecnology, IL, USA). After antibody staining, the blots were stained with Ponceau S (Sigma, Poole, Dorset, UK) to confirm equal protein loading.

### Statistical analysis

Comparisons of irradiated *vs* control DC were carried out by paired *t*-test; pooled cytotoxicity data were compared using an *F* test. The *P*-value was considered statistically significant if *P*⩽0.05. Error bars are s.e.m.

## RESULTS

### Dendritic cells are resistant to radiation-induced apoptosis

**Figure 1:**
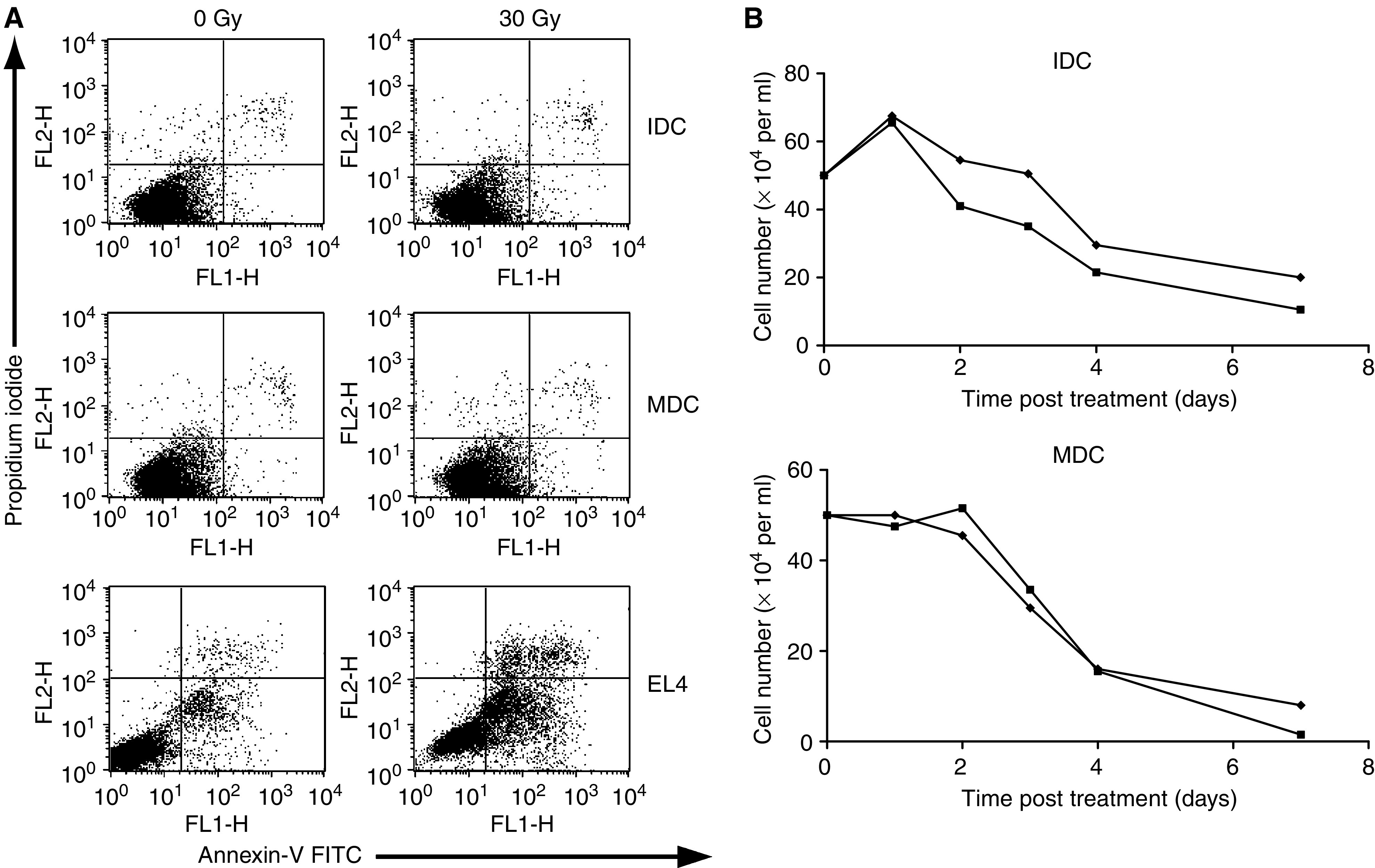
Dendritic cell death following radiation. Apoptosis/necrosis of DC was assessed by FACS analysis after incubation with Annexin-V FITC and propidium iodide (**A**). No significant difference was seen in the apoptotic (lower right quadrant) or necrotic (upper right quadrant) populations of IDC or MDC±30 Gy at 24 h post treatment, in contrast to the significant early apoptotic death of radiosensitive EL4. (**B**) Dendritic cell viability was further assessed by seeding cells at constant density, and counting live cells daily using trypan blue exclusion. No statistical difference was found in the rate of cell death between irradiated (▪) or nonirradiated (⧫) controls for IDC or MDC. Data shown are representative of five similar experiments.

Figure 1A shows the radiosensitivity of immature and mature human DC cultured from CD14^+^ precursors at 24 h following a single 30 Gy treatment. Annexin/PI staining reveals no significant changes in apoptotic (Annexin +ve, PI −ve; lower right quadrant), or necrotic (Annexin +ve, PI +ve; upper right quadrant) populations with irradiation, showing that DC do not undergo a rapid interphase death. As a positive control, extensive apoptotic death post-radiation of the murine lymphoma cell line EL4 is also shown. Figure 1B confirms this resistance of DC over longer time periods by trypan blue exclusion. Immature DC show a transient expansion in number over the first 24 h of culture but then, as anticipated, all differentiated DC lose viability over time. While in this experiment IDC cell loss was slightly faster on irradiation, pooled data from five donors showed no statistically significant difference in the rate of cell death between irradiated and untreated cultures of either MDC or IDC (data not shown). Similar resistance was seen at lower doses (2 and 8 Gy – data not shown). These data show that differentiated DC divide little and do not rapidly apoptose following radiation.

### Irradiated DC show limited changes in surface phenotype, but are less stimulatory in an allogeneic MLR

**Figure 2:**
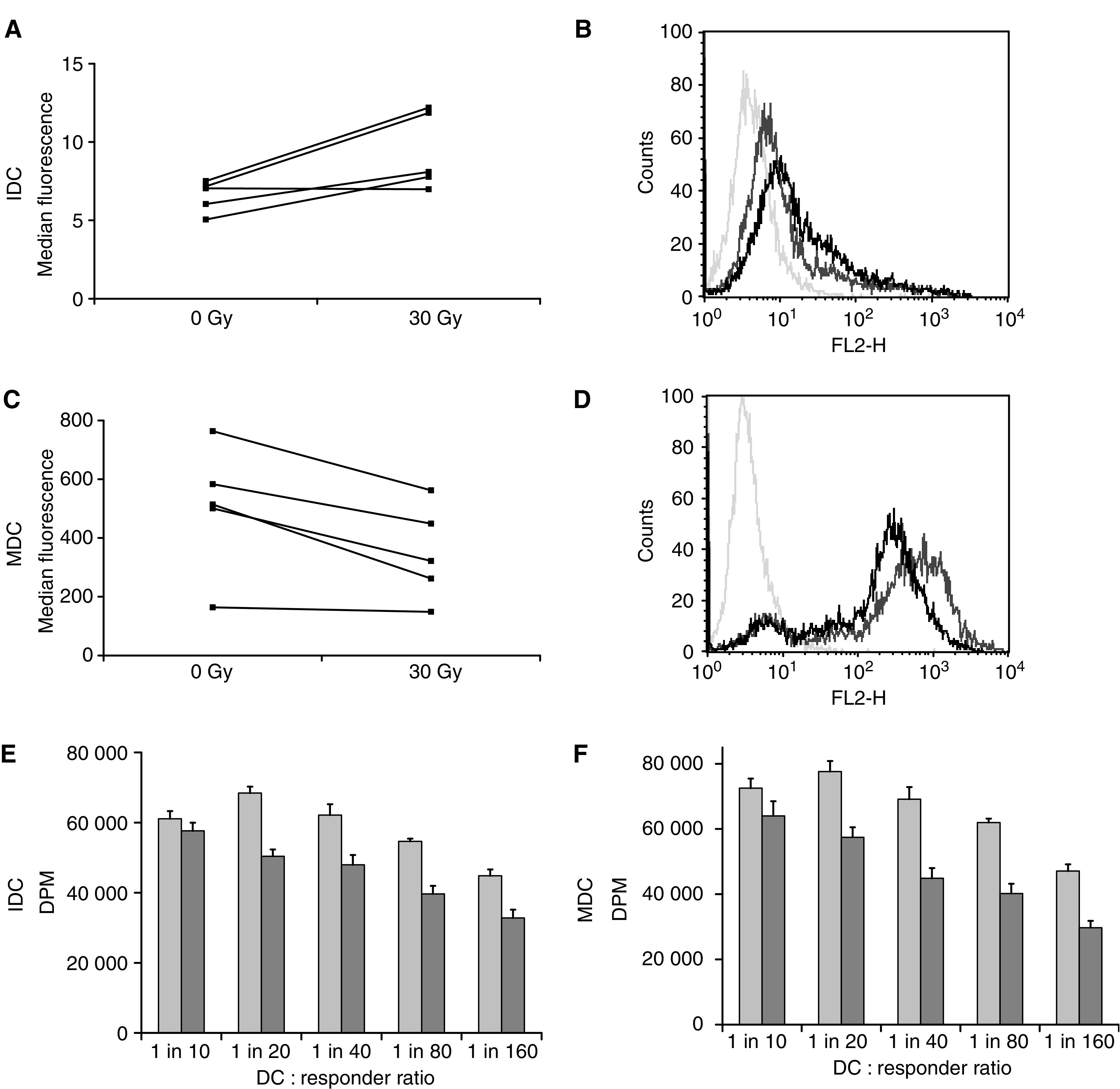
CD86 expression and MLR activity of irradiated dendritic cells. Changes in surface phenotype of DC were assessed 24 h post treatment by FACS analysis. Expression of the co-stimulatory molecule CD86 was significantly upregulated on IDC after irradiation (*P*<0.04), and downregulated on MDC (*P*<0.02), though levels on remained >10 fold less on IDC *vs* MDC. Data shown as MFI of five donors (**A**, **C**), and as a representative histogram for one donor (**B**, **D**). Mixed lymphocyte reaction: irradiated (dark bars) or control (light bars) IDC (**E**) and MDC (**F**) were incubated with responder allogeneic T cells at different ratios and incubated for 5 days before the addition of ^3^H-thymidine for 18 h, followed by harvesting and counting. Data are representative of four similar experiments.

We next studied the surface phenotype of IDC and MDC, with and without irradiation. A range of surface markers for DC maturational state was examined, comprising MHC class I and II, CD40, CD80, CD86, CD83, CD1a and CCR7. The only reproducible changes seen were in surface levels of CD86, after irradiation to 30 Gy. As shown in Figure 2, basal levels of CD86 on IDC were increased with irradiation (although absolute levels remained low), while high CD86 expression on MDC was decreased following 30 Gy. Due to the variable DC phenotype characteristic of individual human donors, data are shown as change in median fluorescence for five separate donors (A and C), and as a representative histogram for one donor (B and D). No significant alteration was seen in the expression pattern of (i) any other marker on IDC or MDC at doses of 2, 8 and 30 Gy, or (ii) CD86 at doses less than 30 Gy (data not shown). These limited changes in surface phenotype of DC on irradiation are consistent with a recent report on irradiation of murine DC (Liao *et al*, 2004).

Since the ability of DC to stimulate proliferation of allogeneic T cells is often used as a surrogate marker of their activation state, we next assessed the potency of irradiated DC in a standard allogeneic MLR. Irradiation of both IDC and MDC to 30 Gy significantly reduced T-cell proliferation in these assays over a range of stimulator : responder ratios, consistent with impaired DC/T-cell interaction following DC radiation (Figure 2E and F). This reduction is consistent with less co-stimulatory CD86 on irradiated MDC, but occurs despite increased (though still relatively low) levels of CD86 on IDC. This suggests that factors other than CD86 expression are involved in determining the potency of DC in an MLR, and implies that irradiation may compromise DC/T-cell interactions during immune priming.

### Irradiated DC largely maintain their endocytic/phagocytic and migratory capacity

**Figure 3:**
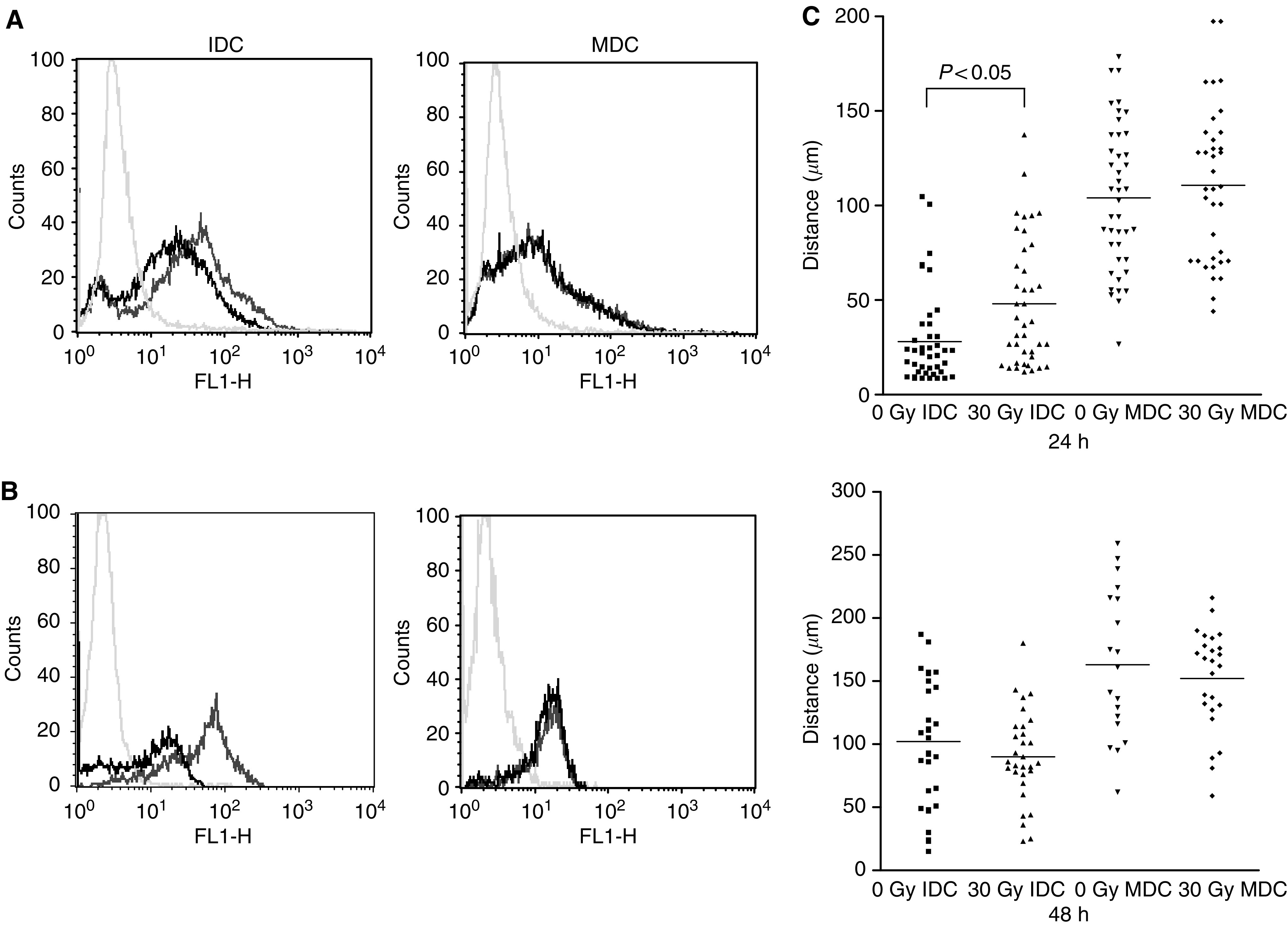
Endocytosis, phagocytosis and motility of irradiated DC. Fluid-phase endocytosis by DC was measured following incubation with BODIPY-labelled BSA (**A**). Dendritic cell were incubated with the dye for 30 min at either 37°C or at 0°C as a negative control (pale grey histogram). Uptake shown by shift in fluorescence for unirradiated DC (dark grey histogram) *vs* irradiated DC (black histogram). Phagocytosis was similarly measured by uptake of fluorescently labelled whole tumour cells, with untreated DC alone as the control (**B**). These data are representative of three similar experiments. (**C**) Tracking (30-min) of IDC and MDC 24 and 48 h post irradiation. Each data point represents the distance travelled by an individual cell, with the horizontal bar showing the mean for the group.

We next examined the endocytic, phagocytic and migratory capacity of irradiated *vs* unirradiated DC, to test whether radiation is likely to effect antigen uptake by DC, or their ability to migrate from sites of antigen acquisition to lymph nodes for interaction with reactive T cells. High endocytic/phagocytic capacity is characteristic of IDC, while greater motility is a feature of MDC. Figure 3A shows fluid phase endocytosis by DC of albumin labelled with BODIPY, a fluorescent dye which is quenched extracellularly and hence specifically demonstrates cell uptake. Fluorescence levels were slightly reduced (though still high) on irradiation of IDC, while 30 Gy did not affect uptake by MDC. When phagocytic capacity by irradiated DC was assessed using uptake of fluorescently labelled human SW480 tumour cells, there was again reduced (though still significant) uptake on irradiation by IDC, and no change for MDC (Figure 3B). Hence antigen uptake by MDC, and to a lesser extent IDC, is largely maintained following irradiation.

To study the *in vitro* migratory capacity of human DC, we quantified the motility of IDC and MDC on fibronectin-coated glass-bottomed dishes. Consistent with published data (De Vries *et al*, 2003), we found increased migration of MDC compared to IDC at 24 and 48 h with or without irradiation (Figure 3C). The only significant difference in the distance DC travelled after radiation was an increase in migration of IDC at 24 h, though this effect was lost at 48 h. MDC motility was unaffected by irradiation at either time point. Overall, these data suggest that irradiated DC will be effective at antigen acquisition and migration to lymph nodes for potential interaction with T cells.

### Irradiated mature DC secrete less IL-12, but maintain IL-10 production

**Figure 4:**
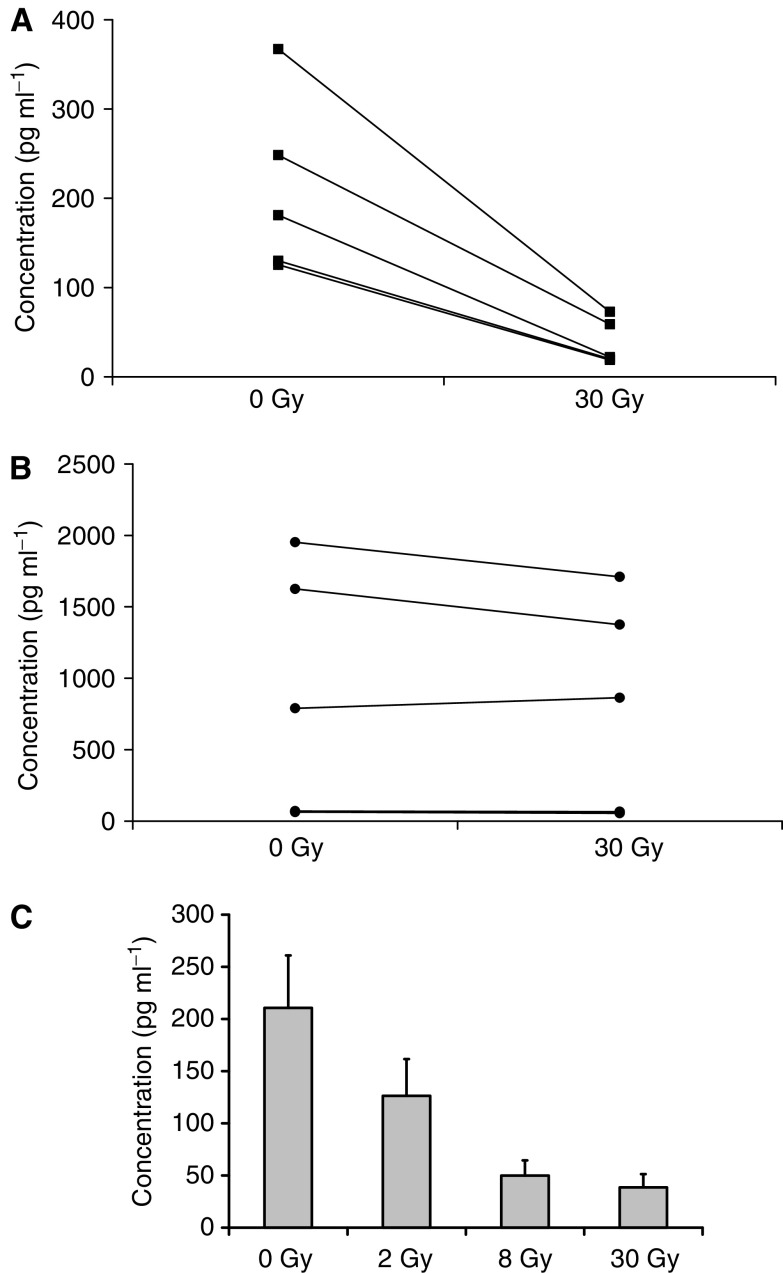
Cytokine secretion by activated DC on irradiation. Cytokine secretion by LPS-activated DC was assayed by ELISA. IL-12 p70 secretion was significantly reduced on irradiation to 30 Gy of DC from five separate donors (*P*<0.001) (**A**), while IL-10 was unaffected (**B**). Pooled results show a dose response in IL-12 secretion at 2, 8, and 30 Gy (**C**).

The cytokines produced by activated DC are key determinants in the generation of T-cell-mediated immunity. Therefore, we measured the cytokines produced by irradiated or unirradiated DC matured with LPS. No cytokines were detected from unstimulated IDC, with or without irradiation (data not shown). Figure 4 shows that MDC secrete both IL-12 (a Th1 cytokine) and IL-10 (Th2) on activation, consistent with previous reports (Qi *et al*, 2003). However, the IL-12/IL-10 balance was different after DC irradiation, with IL-12 production significantly reduced (Figure 4A), while IL-10 was maintained (Figure 4B). The same impaired secretion of IL-12, but not IL-10, was seen when OK432 (Nakahara *et al*, 2003) was used as an alternative maturation factor (data not shown). Since cytokine production by activated human DC can again be variable between donors, Figure 4 illustrates the data for five separate donors. Other cytokines tested (IL-2, IL-4, IL-6, TNF-*α*, IFN-*γ*) were no different between irradiated and unirradiated MDC (data not shown). This shift in IL-12/IL-10 production by activated DC after irradiation may affect the Th1/Th2 balance of the immune response and potentially compromise antitumour therapy (Clerici *et al*, 1998; Lappin and Campbell, 2000).

We further examined the impairment in IL-12 production after irradiation of LPS-matured DC over a range of radiation doses. Figure 4C shows that over five donors, IL-12 production was significantly impaired at a dose as low as 2 Gy. This falls within the range of clinical radiotherapy treatment, and increases the possibility that endogenous DC within treatment fields may be functionally affected in patients.

### Irradiation of peptide-pulsed mature DC impairs naive CTL priming

**Figure 5:**
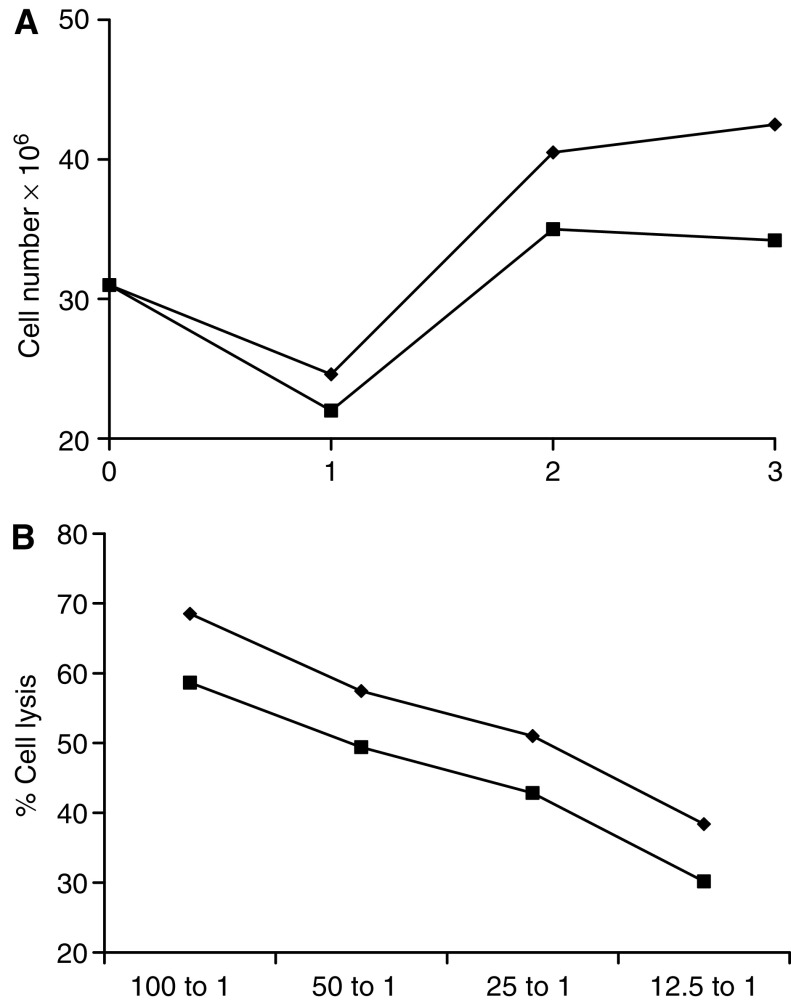
Priming of a naïve cytotoxic T-cell response by irradiated DC. Irradiated (30 Gy) or control MDC were pulsed with MART-1 peptide and incubated with PBMC. T-cell cultures were re-stimulated twice at weekly intervals and assayed for CTL activity at 21 days. Weekly cell counts showed less proliferation of T cells incubated with irradiated (▪) than unirradiated (⧫) DC (**A** shows a representative donor). CTL lysis of peptide-pulsed targets was lower in cultures primed with irradiated DC (square), than untreated controls (diamond) (**B**). These data are shown as pooled results of CTL killing of MART-1 pulsed targets for four separate donors. Unirradiated *vs* irradiated groups were statistically significant (*P*<0.001).

To more rigorously test the consequences of irradiation on DC function, we next examined naïve T-cell priming against a defined tumour-associated antigen, MART-1, using peptide-pulsed HLA-A2+ve MDC as the APC (Gattoni-Celli and Cole, 1996); IDC without activation are ineffective at such priming (Jonuleit *et al*, 2000). This assay directly tests the ability of DC to activate and expand a specific effector T-cell population able to lyse antigen-expressing targets. As shown in Figure 5, MART-1-pulsed MDC irradiated to 30 Gy (a routine procedure in such priming protocols) were able to prime an antigen-specific naïve CTL response. However, when unirradiated MDC were used in these cultures, more T cells proliferated over the 3-week period of culture to generate a greater number of effector cells (Figure 5A). Moreover, these CTL showed higher levels of cytotoxicity, on a per cell basis, against peptide-pulsed targets. While the absolute difference in an individual experiment at any effector : target ratio between lysis by CTL primed with irradiated *vs* control DC was in the region of 10%, this difference proved reproducible and statistically significant across four separate donors (pooled data shown in Figure 5B). The percentage target lysis of control cells pulsed with an irrelevant or no peptide was always <30% at highest 100 : 1 effector : target ratio (data not shown). These data suggest that, when using DC as the APC for optimal priming of naive T cells, irradiation impairs both the number and cytotoxicity of the effector cells generated.

### Irradiation of DC does not effect nuclear translocation of RelB

**Figure 6:**
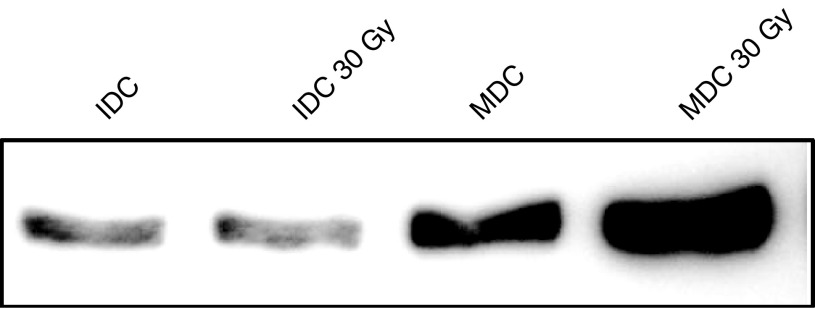
Nuclear RelB expression by irradiated dendritic cells. Nuclear extracts were prepared from irradiated and control IDC and MDC, and RelB expression analysed by Western blot.

The NF-*κ*B/Rel family of proteins are implicated both in the activation of DC, and in the cellular response to radiation (Rescigno *et al*, 1999; Jung and Dritschilo, 2001). To explore the mechanism underlying the effects of radiation on DC, we assessed nuclear translocation of RelB as a marker of DC activation (Pettit *et al*, 1997; Oyama *et al*, 1998). As expected, nuclear RelB was increased in MDC compared to IDC (Figure 6). Irradiation did not effect RelB expression in IDC, but interestingly further increased levels in MDC. Hence, suppression of DC function by irradiation is not due to interference with nuclear translocation of transcription factors of the NF-*κ*B pathway on activation.

## DISCUSSION

The potential role for DC in tumour immunotherapy is rapidly expanding in preclinical studies and early patient trials (Timmerman and Levy, 1999). Both *in vitro* applications of DC and *in vivo* immunotherapy strategies can involve treatment of DC with ionising radiation, often to high dose. The rationale for *ex vivo* DC irradiation is not always clear, but may be designed to prevent continued DC division or the outgrowth of tumour cell components of vaccine preparations (Gong *et al*, 1997; Celluzzi and Falo, 1998; Szmania *et al*, 2001). *In vivo* irradiation of DC may be incidental during clinical radiotherapy, or part of sequencing combined modality treatment with immunotherapy (Teitz-Tennenbaum *et al*, 2003). Regardless of the *in vitro* or *in vivo* scenario, activation of the immune response by irradiated DC may be significantly altered. Although UV is known to suppress the function of some DC subsets (Schmitt and Ullrich, 2000; Denfeld *et al*, 2001; Simon *et al*, 2002; Seite *et al*, 2003), the effects of ionising radiation have not been fully elucidated, and are often assumed to be immunologically neutral.

This study has shown that irradiation of DC significantly affects their behaviour and functional interaction with responding T cells. Our first observation was that DC were resistant to radiation, even following a single high-dose fraction of 30 Gy (Figure 1). It should be noted that in this context resistance applies to radiation-induced apoptosis and early death, rather than later potential mitotic cell death as described for dividing cells, with loss of clonogenicity (Cohen-Jonathan *et al*, 1999). Some increase in loss of IDC was seen over time with irradiation, though this was not statistically significant, and MDC lost viability at identical rates with or without treatment. Annexin/PI staining showed no evidence of the early, interphase apoptosis characteristic of lymphocyte populations (Illidge, 1998). This resistance may reflect the differentiated state of DC which, apart from IDC over the first 24 h, do not proliferate significantly following their initial culture period. While the mechanisms underlying the effects of radiation on DC require further exploration, these data show that any functional changes seen in irradiated DC are not simply due to rapid cell death.

To characterise the phenotype and function of irradiated human DC, we used several *in vitro* assays. Initial experiments measuring surface expression of DC activation markers revealed limited changes only. No significant changes were seen in a panel of surface molecules apart from the co-stimulatory molecule CD86, whose expression was increased on irradiated IDC, and decreased on irradiated MDC (Figure 2A–D). This could imply decreased T-cell stimulatory capacity for irradiated MDC and perhaps some increase for IDC, although absolute CD86 levels on IDC remained very low even after 30 Gy (MFI range 5–12 for IDC, compared to 164–764 for MDC). On testing in an allogeneic MLR, treatment of both IDC and MDC led to impairment of T-cell proliferation (Figure 2E and F), confirming that irradiation impairs T-cell stimulation by DC, and that factors other than CD86 are involved in this defective interaction.

Specific T-cell responses against TAA critically depend on the ability of DC to pick up antigen from their environment and subsequently track to lymph nodes on activation. We therefore tested the endocytic and phagocytic capacities of irradiated DC, as well as their motility on fibronectin-coated plates. For IDC, we found a limited reduction in endocytic and phagocytic capacities and a transient increase in motility on irradiation, while MDC function was unaffected in these assays (Figure 3). Expression of CCR7 (a chemokine receptor upregulated on DC activation to facilitate access to lymph nodes) was unaltered by irradiation of either IDC or MDC (data not shown). This suggests that immune priming by irradiated DC is unlikely to be significantly affected by their ability to acquire antigen or access reactive T cells in lymph nodes.

Although irradiated DC should access T cells in lymph nodes, additional factors are likely to contribute to the final outcome of DC/T-cell interaction. DC-derived cytokines are particularly important in driving a Th1 or Th2 T-cell response, and directing subsequent immunity towards a cytotoxic or regulatory T-cell bias (Lappin and Campbell, 2000). We therefore tested cytokine production in response to DC activation. As shown in Figure 4, the significant change seen in cytokines on DC irradiation was a reduction in levels of secreted IL-12. Since IL-12 is a key factor in priming Th1 responses (Trinchieri, 2003), and Th2 cytokines such as IL-10 were unaltered, this may influence the Th1/Th2 balance of immunity primed by irradiated *vs* unirradiated DC. To explore this hypothesis further, detailed characterisation of the Th phenotype of T cells activated by irradiated DC is currently underway in our laboratory. Interestingly, the reduction in IL-12 production by irradiated DC was seen at doses as low as 2 Gy (Figure 4C), that is, within the range of daily doses used for clinical therapy. If wide fields are used during treatments lasting several weeks, this increases the likelihood that patient DC function may be significantly affected during radiotherapy.

A critical requirement for DC-stimulated immunity in many antitumour therapeutic strategies is the generation of an effective CTL response against defined tumour-associated antigens. CTL represent a key end point in many protocols which currently employ irradiated DC as APC, both *in vitro* and *in vivo* (Gong *et al*, 1997; Celluzzi and Falo, 1998; Fields *et al*, 1998; Szmania *et al*, 2001). Using MART-1 presented by HLA-A2 as a model antigen, we have shown that irradiated human DC prime fewer T cells which are less effective at lysing peptide-pulsed targets (Figure 5). Clearly, irradiation of DC does not entirely prevent CTL priming, as significant cytotoxicity was still seen in cultures using irradiated DC as APC. However, unirradiated DC primed CTL which were significantly more effective at target lysis in multiple donors, and across all effector : target ratios tested (Figure 5B). Hence, when using DC to prime T cells for antitumour immunity, irradiation of DC may impair, though not eradicate, CTL generation.

Activation of the NF-*κ*B pathway, including nuclear translocation of the RelB subunit transcription factor, is a central component of DC activation, and has also been implicated in the cellular response to radiation (Rescigno *et al*, 1999). We therefore tested the effects of DC irradiation on nuclear Rel B expression. Figure 6 shows that enhanced Rel B translocation in MDC compared to IDC was unaffected by irradiation, and that expression levels were even higher in treated MDC than unirradiated controls. This suggests that potential mechanisms underlying suppression of DC function with radiation lie downstream of NF-*κ*B activation, and we are currently further characterising these pathways.

In summary, our data show that irradiation of human DC has important functional consequences, with differential effects on DC at different stages of activation. Radiation of immature DC led to only limited changes in CD86 expression, endocytic/phagocytic activity and early motility. Although irradiated IDC were significantly less potent in an MLR, their ability to tolerise (which has therapeutic implications for the treatment of, for example, autoimmune disease) is currently unknown. Regarding the role of MDC in cancer immunotherapy, this work suggests that irradiation of cultured DC should be avoided if maximal cytotoxic/Th1 T-cell activation is to be realised. The consequences of irradiating endogenous DC *in vivo* during routine therapy or immunotherapy protocols may also be significant, although further studies using clinical material, freshly isolated DC subsets and additional DC populations involved in immune priming (such as plasmacytoid DC) are required.

## References

[bib1] Anton D, Dabadghao S, Palucka K, Holm G, Yi Q (1998) Generation of dendritic cells from peripheral blood adherent cells in medium with human serum. Scand J Immunol 47: 116–121 9496685 10.1046/j.1365-3083.1998.00284.x

[bib2] Banchereau J, Steinman RM (1998) Dendritic cells and the control of immunity. Nature 392: 245–252 9521319 10.1038/32588

[bib3] Cao MD, Chen ZD, Xing Y (2004) Gamma irradiation of human dendritic cells influences proliferation and cytokine profile of T cells in autologous mixed lymphocyte reaction. Cell Biol Int 28: 223–228 14984749 10.1016/j.cellbi.2003.12.006

[bib4] Celluzzi CM, Falo LD (1998) Physical interaction between dendritic cells and tumour cells results in an immunogen that induces protective and therapeutic tumour rejection. J Immunol 160: 3081–3085 9531260

[bib5] Chakravarty PK, Alfieri A, Thomas EK, Beri V, Tanaka KE, Vikram B, Guha C (1999) Flt3-ligand administration after radiation therapy prolongs survival in a murine model of metastatic lung cancer. Cancer Res 59: 6028–6032 10626784

[bib6] Clerici M, Shearer GM, Clerici E (1998) Cytokine dysregulation in invasive cervical carcinoma and other human neoplasias: time to consider the TH1/TH2 paradigm. J Natl Cancer Inst 90: 261–263 9486806 10.1093/jnci/90.4.261

[bib7] Cohen-Jonathan E, Bernhard EJ, McKenna WG (1999) How does radiation kill cells? Curr Opin Chem Biol 3: 77–83 10021401 10.1016/s1367-5931(99)80014-3

[bib8] De Vries IJ, Krooshoop DJ, Scharenborg NM, Lesterhuis WJ, Diepstra JH, Van Muijen GN, Strijk SP, Ruers TJ, Boerman OC, Oyen WJ, Adema GJ, Punt CJ, Figdor CG (2003) Effective migration of antigen-pulsed dendritic cells to lymph nodes in melanoma patients is determined by their maturation state. Cancer Res 63: 12–17 12517769

[bib9] Denfeld RW, Hara H, Tesmann JP, Martin S, Simon JC (2001) UVB-irradiated dendritic cells are impaired in their APC function and tolerize primed Th1 cells but not naive CD4+ T cells. J Leukoc Biol 69: 548–554 11310840

[bib10] Fields RC, Shimizu K, Mule JJ (1998) Murine dendritic cells pulsed with whole tumour lysates mediate potent antitumour immune responses *in vitro* and *in vivo*. Proc Natl Acad Sci USA 95: 9482–9487 9689106 10.1073/pnas.95.16.9482 PMC21364

[bib11] Gattoni-Celli S, Cole DJ (1996) Melanoma-associated tumour antigens and their clinical relevance to immunotherapy. Semin Oncol 23: 754–758 8970598

[bib12] Gong J, Chen D, Kashiwaba M, Kufe D (1997) Induction of antitumour activity by immunization with fusions of dendritic and carcinoma cells. Nat Med 3: 558–561 9142127 10.1038/nm0597-558

[bib13] Guermonprez P, Valladeau J, Zitvogel L, Thery C, Amigorena S (2002) Antigen presentation and T cell stimulation by dendritic cells. Annu Rev Immunol 20: 621–667 11861614 10.1146/annurev.immunol.20.100301.064828

[bib14] Ho WY, Blattman JN, Dossett ML, Yee C, Greenberg PD (2003) Adoptive immunotherapy: engineering T cell responses as biologic weapons for tumor mass destruction. Cancer Cell 3: 431–437 12781360 10.1016/s1535-6108(03)00113-2

[bib15] Illidge TM (1998) Radiation-induced apoptosis. Clin Oncol (R Coll Radiol) 10: 3–13 9543608 10.1016/s0936-6555(98)80104-0

[bib16] Jonuleit H, Giesecke A, Kandemir A, Paragnik L, Knop J, Enk AH (2000) Induction of tumor peptide-specific cytotoxic T cells under serum-free conditions by mature human dendritic cells. Arch Dermatol Res 292: 325–332 10966056 10.1007/s004030000144

[bib17] Jung M, Dritschilo A (2001) NF-kappa B signaling pathway as a target for human tumor radiosensitization. Semin Radiat Oncol 11: 346–351 11677659 10.1053/srao.2001.26034

[bib18] Lappin MB, Campbell JD (2000) The Th1–Th2 classification of cellular immune responses: concepts, current thinking and applications in haematological malignancy. Blood Rev 14: 228–239 11124110 10.1054/blre.2000.0136

[bib19] Liao YP, Wang CC, Butterfield LH, Economou JS, Ribas A, Meng WS, Iwamoto KS, McBride WH (2004) Ionizing radiation affects human MART-1 melanoma antigen processing and presentation by dendritic cells. J Immunol 173: 2462–2469 15294960 10.4049/jimmunol.173.4.2462

[bib20] McBride WH, Chiang CS, Olson JL, Wang CC, Hong JH, Pajonk F, Dougherty GJ, Iwamoto KS, Pervan M, Liao YP (2004) A sense of danger from radiation. Radiat Res 162: 1–19 15222781 10.1667/rr3196

[bib21] Melcher A, Bateman A, Harrington K, Ahmed A, Gough M, Vile R (2002) Dendritic cells for the immunotherapy of cancer. Clin Oncol (R Coll Radiol) 14: 185–192 12109820 10.1053/clon.2001.0038

[bib22] Milas L, Mason KA, Ariga H, Hunter N, Neal R, Valdecanas D, Krieg AM, Whisnant JK (2004) CpG oligodeoxynucleotide enhances tumor response to radiation. Cancer Res 64: 5074–5077 15289307 10.1158/0008-5472.CAN-04-0926

[bib23] Nakahara S, Tsunoda T, Baba T, Asabe S, Tahara H (2003) Dendritic cells stimulated with a bacterial product, OK-432, efficiently induce cytotoxic T lymphocytes specific to tumor rejection peptide. Cancer Res 63: 4112–4118 12874015

[bib24] Oyama T, Ran S, Ishida T, Nadaf S, Kerr L, Carbone DP, Gabrilovich DI (1998) Vascular endothelial growth factor affects dendritic cell maturation through the inhibition of nuclear factor-kB activation in haemopoietic progenitor cells. J Immunol 160: 1224–1232 9570538

[bib25] Pettit AR, Quinn C, MacDonald KPA, Cavanagh LL, Thomas G, Townsend W, Handel M, Thomas R (1997) Nuclear localization of RelB is associated with effective antigen-presenting cell function. J Immunol 159: 3681–3691 9378953

[bib26] Pickl WF, Majdic O, Kohl P, Stockl J, Riedl E, Scheinecker C, Bello-Fernandez C, Knapp W (1996) Molecular and functional characteristics of dendritic cells generated from highly purified CD14+ peripheral blood monocytes. J Immunol 157: 3850–3859 8892615

[bib27] Qi H, Denning TL, Soong L (2003) Differential induction of interleukin-10 and interleukin-12 in dendritic cells by microbial toll-like receptor activators and skewing of T-cell cytokine profiles. Infect Immun 71: 3337–3342 12761116 10.1128/IAI.71.6.3337-3342.2003 PMC155753

[bib28] Rescigno M, Granucci F, Citterio S, Foti M, Ricciardi-Castagnoli P (1999) Coordinated events during bacteria-induced DC maturation. Immunol Today 20: 200–203 10322296 10.1016/s0167-5699(98)01427-3

[bib29] Rescigno M, Winzler C, Delia D, Mutini C, Lutz M, Ricciardi-Castagnoli P (1997) Dendritic cell maturation is required for initiation of the immune response. J Leukoc Biol 61: 415–421 9103227

[bib30] Romani N, Reider D, Heuer M, Ebner S, Kampgen E, Eibl B, Niederwieser D, Schuler G (1996) Generation of mature dendritic cells from human blood. An improved method with special regard to clinical applicability. J Immunol Methods 196: 137–151 8841452 10.1016/0022-1759(96)00078-6

[bib31] Schmitt DA, Ullrich SE (2000) Exposure to ultraviolet radiation causes dendritic cells/macrophages to secrete immune-suppressive IL-12p40 homodimers. J Immunol 165: 3162–3167 10975830 10.4049/jimmunol.165.6.3162

[bib32] Schreiber E, Matthias P, Muller MM, Schaffner W (1989) Rapid detection of octamer binding proteins with ‘mini-extracts’, prepared from a small number of cells. Nucleic Acids Res 17: 6419 2771659 10.1093/nar/17.15.6419 PMC318318

[bib33] Seite S, Zucchi H, Moyal D, Tison S, Compan D, Christiaens F, Gueniche A, Fourtanier A (2003) Alterations in human epidermal Langerhans cells by ultraviolet radiation: quantitative and morphological study. Br J Dermatol 148: 291–299 12588382 10.1046/j.1365-2133.2003.05112.x

[bib34] Simon JC, Hara H, Denfeld RW, Martin S (2002) UVB-irradiated dendritic cells induce nonproliferating, regulatory type T cells. Skin Pharmacol Appl Skin Physiol 15: 330–334 12239427 10.1159/000064537

[bib35] Steinman RM, Hawiger D, Nussenzweig MC (2003) Tolerogenic dendritic cells. Annu Rev Immunol 21: 685–711 12615891 10.1146/annurev.immunol.21.120601.141040

[bib36] Szmania S, Galloway A, Bruorton M, Musk P, Aubert G, Arthur A, Pyle H, Hensel N, Ta N, Lamb Jr L, Dodi T, Madrigal A, Barrett J, Henslee-Downey J, van Rhee F (2001) Isolation and expansion of cytomegalovirus-specific cytotoxic T lymphocytes to clinical scale from a single blood draw using dendritic cells and HLA-tetramers. Blood 98: 505–512 11468143 10.1182/blood.v98.3.505

[bib37] Teitz-Tennenbaum S, Li Q, Rynkiewicz S, Ito F, Davis MA, McGinn CJ, Chang AE (2003) Radiotherapy potentiates the therapeutic efficacy of intratumoral dendritic cell administration. Cancer Res 63: 8466–8475 14679011

[bib38] Timmerman JM, Levy R (1999) Dendritic cell vaccines for cancer immunotherapy. Annu Rev Med 50: 507–529 10073291 10.1146/annurev.med.50.1.507

[bib39] Trinchieri G (2003) Interleukin-12 and the regulation of innate resistance and adaptive immunity. Nat Rev Immunol 3: 133–146 12563297 10.1038/nri1001

[bib40] Yee C, Thompson JA, Roche P, Byrd DR, Lee PP, Piepkorn M, Kenyon K, Davis MM, Riddell SR, Greenberg PD (2000) Melanocyte destruction after antigen-specific immunotherapy of melanoma: direct evidence of t cell-mediated vitiligo. J Exp Med 192: 1637–1644 11104805 10.1084/jem.192.11.1637 PMC2193107

